# Prevalence and clinical characteristics of T2DM patients with OTUD3 gene rs78466831 SNP at a single academic center in China

**DOI:** 10.3389/fendo.2022.1059641

**Published:** 2022-12-02

**Authors:** Jian-Ping Liu, Ai-Ping Yang, Gang Lei, Man Yu, Yu Peng, Ai-ping Le

**Affiliations:** ^1^ Department of Clinical laboratory, The First Affiliated Hospital of Nanchang University, Nanchang, Jiangxi, China; ^2^ Department of Clinical Laboratory, Shanghai Songjiang Jiuting Hospital, Shanghai, China; ^3^ Department of Critical Care Medicine, People’s Hospital of Zhangshu, Zhangshu, Jiangxi, China; ^4^ Department of Clinical Laboratory, Hubei Cancer Hospital, Wuhan, Hubei, China; ^5^ Department of Ophthalmology, The First Affiliated Hospital of Nanchang University, Nanchang, Jiangxi, China; ^6^ Department of Transfusion, The First Affiliated Hospital of Nanchang University, Nanchang, Jiangxi, China; ^7^ Key Laboratory of Blood Transfusion Medicine of Jiangxi Province, The First Affiliated Hospital of Nanchang University, Nanchang, Jiangxi, China

**Keywords:** OTUD3, type 2 diabetes, clinical characteristics, gene, diabetes retinopathy

## Abstract

**Background:**

A novel, rare OTUD3 c.863G>A (rs78466831) in humans has been reported associated with diabetes, but the prevalence and clinical characteristics of T2DM patients with rs78466831 have not been reported before.

**Objective:**

To investigate the prevalence and clinical characteristics of T2DM patients with rs78466831 and provide a basis for clinical diagnosis and treatment.

**Methods:**

OTUD3 gene rs78466831 SNP was detected by Sanger sequencing in all the collected specimens of laboratory-confirmed T2DM patients and healthy people. Clinical characteristics indexes inconsisting of fasting blood glucose (FBG), glycosylated hemoglobin (HbA1c), high density lipoprotein cholesterol (HDL-C), low density lipoprotein cholesterol (LDL-C), total cholesterol (TC), triglyceride (TG) and a body mass index (BMI), T2DM-associated chronic complications (myocardial infarction, cerebrovascular disease, retinopathy, arterial plaque, peripheral neuropathy and nephropathy) were obtained from the clinical laboratory information systems and electronic medical record system. Clinical characteristic indicators were compared between the wild-type and variant (rs78466831) patients with T2DM.

**Results:**

The prevalence of rs78466831 in the T2DM patients group was significantly higher than the healthy control in our academic center. The general characteristic indicators were not significantly different between the wild-type and rs78466831 patients with T2DM, except the family history of diabetes. Clinical laboratory indicators including HbA1c, FBG, OGTT, TC, HDL-C, LDL-C and CP had no significant difference between the two groups. The therapeutic drug and target achievement rates were not significantly different between the two groups. The incidence of diabetic retinopathy in the variant group was significantly higher than the wild-type group.

**Conclusions:**

The OTUD3 gene rs78466831 was associated with T2DM and may be a biological risk factor of diabetes retinopathy.

## Introduction

The prevalence of type 2 diabetes mellitus (T2DM) has increased worldwide over the past several decades. T2DM is the most common form of diabetes, accounting for more than 90% of diabetes cases in China (1–3). The International Diabetes Federation (IDF) reported that in 2015, more than 400 million adults worldwide suffered from diabetes (4). The IDF estimates that this number will exceed 600 million by 2040. China currently has the largest number of T2DM cases worldwide. T2DM can lead to many different chronic complications that can reduce the quality of life and even induce premature death. T2DM has a multifactorial etiology, and genes play a key role in its pathogenesis (5). For example, Lee et al. found that the Gas6 gene rs8191974 SNP is associated with T2DM cases in Taiwan (6). The Gas6 polymorphism is associated with stroke (7). The Gas6/TAM system is involved in the pathogenic mechanism of diabetes-associated renal and cardiovascular complications (8). Moreover, low levels of AIM2 promoter total methylation might increase the risk of T2DM and AIM2 promoter total methylation or some loss of CpG methylation increase the risk of vascular complications in T2DM (9). Therefore, we can speculate that patients with T2DM may have different clinical characteristics due to various susceptibility genes. Genetic tests can not only reveal clinical subgroups but can also result in improved treatment outcomes for these patients. For example, combined multigene screening before therapy and LDL-C and sdLDL-C detection before and after therapy could well assist T2DM treatment (10). Brown et al. suggested that increased SLC4A4/NBCe1 in β cells in T2DM contributes to the promotion of β cell failure and should be considered as a potential therapeutic target (11).

In 2022, Zhou et al. reported that in humans, the novel, rare OTUD3 c.863G>A (rs78466831) mutation is associated with diabetes (12). They found that the wild-type genotypes in healthy controls were GG and all the variants were heterozygous GA. OTUD3 c.863 G>A reduced protein stability and DUB activity, which is important for the function of OTUD3 in humans. The data of that study suggested that the CREB-binding-protein-dependent OTUD3 (CBP–OTUD3) signaling pathway plays a key role in glucose and fatty acid metabolism. Glucose and fatty acids can stimulate CBP–OTUD3 acetylation, thus promoting nuclear translocation, wherein OTUD3 regulates various genes involved in glucose and lipid metabolism and oxidative phosphorylation by stabilizing peroxisome-proliferator-activated receptor delta (PPARd) (12).

However, the prevalence and clinical characteristics of T2DM patients with rs78466831 in different regions are still unknown. Clinical characteristics can provide useful information for the effective treatment and management of patients who suffer from diabetes. The comparison of clinical laboratory indicators, general characteristics, target achievement rates, selected hypoglycemic drugs, and associated complications needs further investigation. Therefore, in this study, we intend to explore the data from our academic center to provide a basis for clinical diagnosis and treatment.

## Materials and methods

### Patients

Cases of T2DM were diagnosed on basis of the 1999 WHO guidelines (13, 14). Patients with T2DM were diagnosed by two endocrinologists in the in-patient departments of the First Affiliated Hospital of Nanchang University. All patients were admitted voluntarily. The inclusion criterion for the patients with T2DM was either fasting plasma glucose level ≥ 7.0 mmol/L or 2 h oral glucose tolerance test glucose level ≥ 11.1 mmol/L. The exclusion criteria included type 1 diabetes, gestational diabetes mellitus, and other special types. The specimens for laboratory detection were collected from patients with type 2 diabetes who were hospitalized in the First Affiliated Hospital of Nanchang University. Blood samples for diagram construction were donated by one of the variant’s family members with informed consent. The samples of healthy adults were collected in the health examination center of our hospital. The inclusion criteria for healthy adults were as follows: 1. Clinical biochemical tests (liver function test, kidney function test, blood glucose test, and blood lipid test) within the normal reference range. 2. Routine blood test indexes within the normal reference range. 3. Routine urine tests within the normal reference range. The exclusion criteria were as follows: 1. History of diabetes. 2. Family history of T2DM. 3. Systemic diseases. 4. Renal and hepatic failure. 5. Cardiovascular disease. 6. Malignant tumors, infections, or other endocrine diseases. 7. Other types of diabetes.

### DNA extraction and quality control

A DNeasy Blood & Tissue Kit (69506, Qiagen, Germany) was used to extract blood genomic DNA. NanoDrop ND-1000 (Thermo Fisher Scientific, USA) was applied to detect DNA concentration and quality. A A260/A280 ratio between 1.7–2.0 indicated high DNA purity. DNA was diluted to the working solution concentration of 20 ng/µL for further study.

### Amplification

The primers for the detection of the G>A single-nucleotide mutation of the OTUD3 gene (rs78466831 SNP) were designed by the online software Primer-BLAST provided by the National Center for Biotechnology Information. The forward primer sequence of the OTUD3 rs78466831 SNP was GACTGAAGTAGGGACCCAGG, and the reverse primer sequence was ACTGTCACGGCATACACCAA. The length of the amplified fragment was 480 bp. The polymerase chain reaction (PCR) system had the total volume of 40 µL and contained 2 µL of 10 µmol/L Primer F, 2 µL of 10 µmol/L Primer R, 1 µL of 20 ng/µL template gDNA, 20 µL of 2× T8 High-Fidelity Master Mix, and 15 µL of ddH_2_O. The reaction procedure was as follows: 98 °C for 2 min, 35 cycles of 98 °C for 10 s, 58 °C for 10 s, and 72 °C for 15 s then at 72 °C for 5 min. A PCR amplification instrument (A300, LONGGENE, China) was used.

### Sanger sequencing (G–normal allele; A–variant allele)

The amplified PCR products were subjected to agarose gel electrophoresis (2 µL of sample + 6 µL of bromophenol blue) at the voltage of 300 V for 12 min. The gel map showed that the target band size was single. Then, the qualified PCR products were Sanger sequenced by a sequencer (ABI 3730XL, Thermo Fisher Scientific). The rs78466831 SNP was analyzed by using Sequencing Analysis 5.2 software.

### Detection of clinical laboratory indicators

Fasting blood glucose (FBG), total cholesterol (TC), triglyceride (TG), low-density lipoprotein cholesterol (LDL-C), high-density lipoprotein cholesterol (HDL-C), and C-peptide were detected by using serum samples. The clinical biochemical indexes of the patients were determined by using a Hitachi 7600 automatic biochemical analyzer. The detection methods were as follows: fasting blood glucose (FBG): hexokinase method; TC: oxidase method; TG: enzymatic method; LDL-C: direct clearance method; and HDL-C: direct clearance method. All the above biochemical testing reagents were provided by Ningbo Meikang Biotechnology Co., LTD. C-peptide was detected through chemiluminescent immunoassay by using *in vitro* diagnostic kits and MAGLUMI chemiluminescence detector were produced by Shenzhen New Industry Biomedical Engineering Co., LTD. Glycosylated hemoglobin (HbA1C) was detected through high-performance liquid chromatography (TOSOH HLC-723G8).

### Collection of general characteristic data

General characteristic information, such as sex, age, diagnose age of onset, body mass index (BMI = weight (kg)/(height [m]^2^),blood pressure, smoking, drinking, associated chronic complications, family history of diabetes and hypoglycemic drug use of T2DM patients were collected through the Clinical electronic medical record system. And some incomplete medical records were supplemented by telephone questionnaires. The T2DM-associated chronic complications of the patients were judged by the diagnosis medical records and the abnormal results of diagnostic examinations. The patients were assumed to have CHD (coronary heart disease) if they had been diagnosed by the diagnostic examinations included coronary angiography or coronary artery computed tomography. Retinopathy was diagnosed according to the ophthalmologic test, arterial plaque was diagnosed by carotid ultrasonography and diabetic nephropathy was diagnosed as GFR (glomerular filtration rate) <60 mL/min/1.73 m^2^ or urinary albumin to creatinine ratio>30 mg/g. Peripheral Neuropathy was diagnosed by Neuroelectrophysiological examination.

### Therapeutic drug and target value achievement rate of patients with T2DM on admission

The standard treatment of the patients was based on the 2017 China guidelines for T2DM (14). The target values of treatment were set as follows: FBG 4.4–7.0 mmol/L, HbA1c < 7.0%, TC < 4.5 mmol/L, TG < 1.7 mmol/L, HDL‐C > 1.0 mmol/L (men) or >1.3 mmol/L (women), LDL‐C < 2.6 mmol/L (not accompanied by CHD) or <1.8 mmol/L (accompanied by CHD), and BMI < 24 kg/m^2^.

### Statistical analysis

Data were analyzed by using SPSS 20.0 (SPSS Inc., Chicago, USA). P-values < 0.05 were statistically significant. Continuous variables were descriptively analyzed by using the mean and standard deviation, whereas categorical variables were summarized as counts and percentages in each category. The general characteristics and laboratory indicators were analyzed through t-tests (for normally distributed variables) and Mann–Whitney U test (for non-normally distributed variables). Chi-square test was applied to analyze T2DM-associated complications.

## Results

### Results of the prevalence of rs78466831 in our academic center

We found six variants (rs78466831) in 300 patients with T2DM and zero in the healthy controls (**Table 1**). All the genotypes of the variants were GA. The frequencies of allele A in patients with T2DM and healthy controls were 1% and 0%, respectively. In our academic center, the prevalence of rs78466831 in the patients with T2DM was significantly higher than that in the healthy controls.

**Table 1:** Different prevalence rates of rs78466831 between healthy controls and patients with T2DM in our academic center.

subjects	Genotypes			Allele	
	GG	GA	AA	G	A
Healthy	300	0	0	600	0
T2DM	294	6	0	594	6
OR	1.01				
P	0.041				
95% CI	1.002-1.018				

CI, Confidence Interval.

### Diagram of a variant family

**Figure 1:**
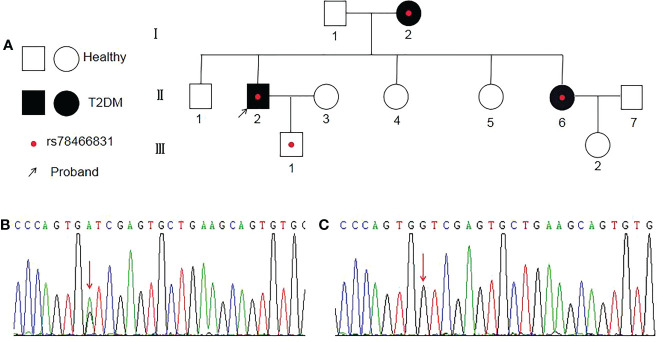
Diagram of a family with rs78466831. **(A)** Proband is indicated with an arrow. ◯ females; □ males; ● female with T2DM; ■ male with T2DM; ● with rs78466831. **(B)** Single-base substitution mutation (rs78466831) is indicated with a red arrow. **(C)** Normal base (red arrow).

All of the variants, except for one child (child-III-1), had type 2 diabetes (**Figure 1A**). One of the three patients in the family had an onset age earlier than 35 years old, and two had an onset age of greater than 35 years old. The genotypes of the patients were heterozygous mutations GA (**Figure 1B**), the other healthy adults were wild-type GG (**Figure 1C**). The arrow indicates the position of the mutant base (rs78466831 position).

### General characteristics of the wild-type and rs78466831 patients with T2DM

Among 300 cases, 148 wild-type cases with complete data were selected as the control group and compared with the variant (rs78466831) group. The general characteristics (**Table 2**), including age, sex (M/F), diagnosed age of onset, diabetes, duration (years), hypertension, smoking (%), and alcohol (%) did not significantly differ between the two groups. However, the family history of diabetes significantly differed between the two groups. The variants all had a family history of diabetes at rates significantly higher than those in the control group.

**Table 2:** General characteristics of the wild-type and rs78466831 patients with T2DM.

Variables	Wide-type	rs78466831	P-Value
Age	59.07± 11.684	58.83±14.497	0.962
Sex(M/F)	94/54	5/1	0.422
Family history	30/148	6/6	<0.01
Diagnose age of onset	49.49±11.2	49.50±18.15	0.999
Diabetes duration (years)	9.57±7.13	9.33±7.9	0.936
Hypertension	101/148	2/6	0.879
Smoking (%)	35/148	3/6	0.16
Alcohol(%)	27/148	1/6	0.921

### Clinical laboratory characteristics of the two groups

Clinical laboratory indicators, including HBA1c, FBG, OGTT, CP, TC, HDL-C, TG, and LDL-C, did not significantly differ between the two groups (**Table 3**).

**Table 3:** Clinical laboratory characteristics of the wild-type and rs78466831 patients with T2DM.

Variables	Wide-type	rs78466831	P-Value
HBA1c(%)	9.103±2.798	8.333±1.405	0.504
FBG (mmol/L)	9.706±5.313	9.21±6.024	0.825
OGTT 1h (mmol/L)	13.353±5.423	10.966±2.116	0.315
OGTT 2h (mmol/L)	14.037±6.274	11.255±2.339	0.263
CP(ng/mL, 0h)	1.1097±1.03559	0.8700±0.922	0.578
CP (ng/mL,1h)	2.107±1.693	1.283±0.851	0.238
CP (ng/mL, 2h)	2.328±1.958	1.36±0.736	0.350
TC(mmol/L)	4.56±1.309	5.505±1.352	0.086
HDL-C (mmol/L)	1.206±0.40524	1.417±0.433	0.214
TG(mmol/L)	1.746±1.637	1.44±0.387	0.649
LDL-C(mmol/L)	2.848±1.175	3.618±1.22	0.118

HbA1c, Glycosylated hemoglobin; FBG, Fasting blood glucose; OGTT, Oral glucose tolerance test; TC, Total serum cholesterol; HDL-C, High‐density lipoprotein cholesterol; HDL-C, Low‐density lipoprotein cholesterol; CP, C peptide.

### The selection of treatment drugs and the target achievement rate of the two groups at admission

In accordance with the 2017 China guidelines for T2DM, the therapeutic drug selections and target value achievement rate (**Table 4**) of the wild-type and rs78466831 patients with T2DM at admission did not significantly differ between the two groups. See the table below for details.

**Table 4:** Therapeutic drug and target value achievement rates of the wild-type and rs78466831 patients with T2DM.

Variables(%)	wide-type	rs78466831	P-value
Oral agents (hypoglycemic drug )	128/148	6/6	0.192
Insulin (hypoglycemic drug )	112/148	6/6	0.337
FBG(4.4‐7.0 mmol)	46/148	3/6	0.383
HbA1c (<7.0%)	42/148	1/6	0.463
TC(<4.5 mmol/L)	75/148	2/6	0.681
HDL-C (>1.0 mmol/L men or >1.3 mmol/L women)	101/148	5/6	0.666
TG(<1.7 mmol/L)	89/148	2/6	0.227
LDL-C(<2.6 mmol/L ,not accompanied by CHD or <1.8 mmol/L, accompanied by CHD))	66/148	1/6	0.234
BMI ( <24 kg/m^2^)	90/148	5/6	0.255

### Comparison of T2DM-associated chronic complications between the two groups

T2DM-associated chronic complications, including nephropathy, cerebrovascular disease, cardiovascular disease, arterial plaque, and peripheral neuropathy did not significantly differ between the two groups (**Table 5**). However, the incidence rate of diabetic retinopathy (DR) was 100% in the variant group and was significantly higher than that in the control group. In most variant cases, punctate hemorrhage and exudation can be seen in the retinas of both eyes.

**Table 5:** T2DM-associated chronic complications of the wild-type and rs78466831 patients with T2DM.

Variables	wide-type	rs78466831	p-value
Retinopathy (%)	32/148	6/6	<0.01
Nephropathy (%)	41/148	2/6	0.616
Cerebrovascular disease (%)	10/148	1/6	0.364
Cardiovascular disease (%)	16/148	0/6	0.246
Arterial plaque (%)	76/148	2/6	0.439
Peripheral Neuropathy (%)	85/148	3/6	0.720

## Discussions

OTU-domain ubiquitin aldehyde-binding proteins (OTUs) are members of DUBs, which can reverse protein ubiquitination (15–17). DUBs are crucial for cellular functions and can be divided into six families, including ubiquitin C-terminal hydrolases, ubiquitin-specific processing proteases, Jab1/Pab1/MPN domain-containing metalloenzymes, OTU Ataxin-3/Josephin, and monocyte chemotactic protein-induced proteases (18). DUBs have been found to regulate many important cellular functions, such as DNA repair, gene expression, cell cycle progression, apoptosis, kinase activation, proteasome or lysosome-dependent protein degradation, and protein degradation prevention (19).

OTUD3 is a hot topic in studies on OTUs. Although OTUD3 has been well described as a key factor in tumorigenesis (20–24), its physiological functions still need further understanding. The variant SNP (rs78466831) found in a MODY-like family is a high-risk factor of diabetes. A novel regulatory mechanism wherein OTUD3 can regulate energy metabolism by blocking ubiquitin-dependent PPARd degradation was found. MODY is easily misdiagnosed as type 2 diabetes because its clinical features always largely overlap with those of type 2 diabetes (25–27). The data of the ALFA project (Release Version: 20201027095038), which provides aggregate allele frequency, showed that the allele frequency of this mutation varies by race and region. The variant allele A frequency of the mutation is significantly higher in East Asian populations (approximately 0.69%) than in other populations (almost zero). Therefore, in our study, we intended to explore the prevalence and clinical characteristics of T2DM patients with rs78466831, including laboratory indicators, age of onset, treatment, complications, and family history of diabetes, from a single academic center.

Our study further confirmed that the rs78466831 mutation was associated with type 2 diabetes in a province located in east China. The general characteristics, including age, sex (M/F), age of onset, duration (years), hypertension, smoking (%), and alcohol (%), but not family history of diabetes, did not significantly differ between the two groups. The variants all had a family history of diabetes at rates significantly higher than those in the control group. The family diagrams showed that all of the variants, except for one child (III-1), had type 2 diabetes. Type 2 diabetes is well known to be an age-related disease that is prevalent only in the adult population. One of the three patients in the family had an onset age earlier than 35 years old, and two had an onset age later than 35 years old. Age of onset may differ due to the varying diets, lifestyles, and environmental factors of individual patients (28). Therefore, we can infer that the rs78466831 gene plays an important role in the development of T2DM on the basis of the family history of diabetes and diagram.

Furthermore, we compared the laboratory characteristics of rs78466831 patients with T2DM with those of the wild-type patients with T2DM. Most laboratory indicators, including HBA1c, FBG, OGTT, CP, TC, HDL-C, TG, and LDL-C, did not significantly differ between the two groups. Therefore, distinguishing patients with rs78466831 on the basis of common laboratory indicators was difficult. Furthermore, the therapeutic drug selected and target value achievement rates did not significantly differ between the two groups on admission. However, the incidence of DR in the variant group was significantly higher than that in the wild-type group. Zhang et al. reported that OTUD3 restricts innate antiviral immune signaling. The acetylation-dependent deubiquitinase OTUD3 controls MAVS activation in innate antiviral immunity. IL-6, Tnf-a, IL-1b, and Nos2, which are critical NF-kB target genes activated by MAVS aggregation, are consistently and efficiently induced by SeV in OTUD3-deficient macrophages (29). Most of the inflammatory cytokines mentioned above have been reported to be associated with DR (30, 31). The positive effect of anti-inflammatory therapeutics in patients with DR have highlighted the central involvement of the innate immune system (32), and immune dysregulation has become increasingly identified as a key element of the pathophysiology of DR by interfering with normal homeostatic systems (33, 34). Therefore, we inferred that OTUD3 c.863 G>A leads to reductions in protein stability and DUB activity, which may result in the impaired function of the innate immune system and the higher frequency of retinopathy in the variant patients than in the wild-type patients. DR is recognized as the leading cause of visual impairment and acquired blindness among adults worldwide (35, 36). The incidence of DR in the wild-type group was consistent with that reported by other scholars. To illustrate, in a meta-analysis of approximately 23 000 people with diabetes worldwide, the prevalence of DR was approximately 36% (37). DR is reported to have genetic and acquired (environmental) factors (38, 39). For example, a study in Japan reported associations between long noncoding RNA RP1-90L14 and susceptibility to DR (40). Therefore, the high prevalence of DR in the variants suggested that rs78466831 may be a risk factor of DR. Additionally, given that DR may be asymptomatic for years even at an advanced stage (41–43), screening is crucial to identify, monitor, and guide the treatment of retinopathy. Currently, the diagnosis of DR status should be based on ophthalmoscopy or mydriatic or nonmydriatic retinal photography (44–46). Therefore, we suggest that T2DM patients with rs78466831 should be regularly screened for DR. This approach may help patients obtain accurate treatment and reduce the harm of DR.

Our research has some limitations. First, we only investigated the prevalence and clinical characteristics of T2DM patients with the OTUD3 gene rs78466831 SNP from a single academic center in China and not from multiple clinical research centers. Therefore, a large study is needed. Such a study may be costly, but important. Furthermore, the exact pathological mechanisms of the OTUD3 gene rs78466831 SNP in DR progression remain unknown. Finally, even if the susceptible gene of DR is identified, alterations in gene expression may occur because of environmental factors. Thus, epigenetics studies are very necessary.

To summarize, T2DM is a heterogeneous and broad-spectrum disease with many variations (47). The clinical characteristics of T2DM subtypes may vary depending on the genetic and environmental background (48–50). Our study confirmed that the mutation rs78466831 is associated with type 2 diabetes in a province located in east China. Most laboratory indicators did not significantly differ between the two groups. However, the incidence of DR in the variant group was significantly higher than that in the wild-type group. Therefore, rs78466831 can be a biomarker of DR. This finding will be helpful for the early treatment and management of DR in such patients.

## Data availability statement

The datasets presented in this study can be found in online repositories. The names of the repository/repositories and accession number(s) can be found in the article/supplementary material.

## Ethics statement

This study had been approved by the Medical Research Ethics Committee of the First Affiliated Hospital of Nanchang University. Ethics No.2020 (8–42). Written informed consent from the participants’ legal guardian/next of kin was not required to participate in this study in accordance with the national legislation and the institutional requirements.

## Author contributions

J-PL conceived the study and wrote the manuscript. A-PL revised the manuscript. A-PY analyzed the data. MY collected the samples and detected indicators levels. GL assisted in specimen collection. YP checked the patient’s clinical data. All authors read and approved the final manuscript.

## Funding

This work is supported by the Science and Technology Innovation Base Plan of Jiangxi province (20212BCD42006), the Key Research and Development Plan Project of Jiangxi Province (20202BBG73013), the Science and Technology Plan project of Education Department of Jiangxi Province(GJJ180134) and the Science and Technology Project of Health and Family Planning Commission of Jiangxi Province of China(20195073).

## Acknowledgments

We thank all the doctors, nurses, researchers and patients who participated in this project. We would like to thank Prof. Jing Zhu for her critical discussion and editing of the manuscript.

## Conflict of interest

The authors declare that the research was conducted in the absence of any commercial or financial relationships that could be construed as a potential conflict of interest.

## Publisher’s note

All claims expressed in this article are solely those of the authors and do not necessarily represent those of their affiliated organizations, or those of the publisher, the editors and the reviewers. Any product that may be evaluated in this article, or claim that may be made by its manufacturer, is not guaranteed or endorsed by the publisher.
